# Mechanical behavior of full-thickness burn human skin is rate-independent

**DOI:** 10.1038/s41598-024-61556-8

**Published:** 2024-05-15

**Authors:** Samara Gallagher, Kartik Josyula, Rahul, Uwe Kruger, Alex Gong, Agnes Song, Emily Eschelbach, David Crawford, Tam Pham, Robert Sweet, Conner Parsey, Jack Norfleet, Suvranu De

**Affiliations:** 1 Department of Mechanical, Aerospace, and Nuclear Engineering, Rensselaer Polytechnic Institute, Troy, NY USA; 2 Center for Modeling, Simulation, and Imaging in Medicine, Rensselaer Polytechnic Institute, Troy, NY USA; 3 Department of Biomedical Engineering, Rensselaer Polytechnic Institute, Troy, NY USA; 4 Center for Research in Education and Simulation Technologies, University of Washington, Seattle, WA USA; 5 UW Medicine Regional Burn Center at Harborview Medical Center, University of Washington, Seattle, WA USA; 6 U.S. Army Combat Capabilities Development Command - Soldier Center, Simulation and Training Technology Center, Orlando, FL USA

**Keywords:** Statistical methods, Biomechanics, Machine learning

## Abstract

Skin tissue is recognized to exhibit rate-dependent mechanical behavior under various loading conditions. Here, we report that the full-thickness burn human skin exhibits rate-independent behavior under uniaxial tensile loading conditions. Mechanical properties, namely, ultimate tensile stress, ultimate tensile strain, and toughness, and parameters of Veronda–Westmann hyperelastic material law were assessed via uniaxial tensile tests. Univariate hypothesis testing yielded no significant difference (p > 0.01) in the distributions of these properties for skin samples loaded at three different rates of 0.3 mm/s, 2 mm/s, and 8 mm/s. Multivariate multiclass classification, employing a logistic regression model, failed to effectively discriminate samples loaded at the aforementioned rates, with a classification accuracy of only 40%. The median values for ultimate tensile stress, ultimate tensile strain, and toughness are computed as 1.73 MPa, 1.69, and 1.38 MPa, respectively. The findings of this study hold considerable significance for the refinement of burn care training protocols and treatment planning, shedding new light on the unique, rate-independent behavior of burn skin.

## Introduction

Skin provides natural protection to the body against external thermomechanical stimuli. The mechanical response of skin is related to its hierarchical structure and properties of collagen fibers, elastin, and the proteoglycans in the skin^1^. Normal skin exhibits rate-dependent behavior, which is attributed to the viscous sliding of collagen fibers within the interstitial fluids and extracellular matrix^2–4^. Thermal treatment has been found to alter the water content and collagen structure in the skin tissue^5–8^, potentially leading to different strain rate sensitivity in thermally damaged skin compared to healthy skin. However, this aspect has not been thoroughly explored in the existing literature. This study aims to investigate the strain rate sensitivity of full-thickness burn ex vivo human skin. Gaining a fundamental understanding of the strain rate sensitivity of thermally injured skin tissue is essential for developing high-fidelity burn care simulators and in designing and delivering improved medical interventions for burn care^9,10^.

Skin tissue can be divided into three layers, i.e., epidermis, dermis, and subcutaneous fat or hypodermis. The topmost epidermal layer is avascular and primarily comprised of several layers of keratinocytes^11^. It is a thin layer with thickness of 60–100 μm, and further divided into stratum basale, stratum spinosum, stratum granulosum, stratum lucidum, and stratum corneum, which is the outermost layer^11^. The keratin and lipid networks in the stratum corneum govern the permeability of skin and the rate dependent response to mechanical loads^12^. The dermal layer constitutes most of the skin with a thickness of 0.6–3 mm^11^. It is primarily made of collagen and elastin fibers within a hydrated proteoglycan matrix, which are responsible for the mechanical behavior, i.e., the elastic properties and strength of the skin tissue^11^. The rate dependent mechanical behavior of the skin is a result of the shear interaction between collagen fibers and the hydrated proteoglycan matrix^13^.

The rate-dependent mechanical behavior of human skin tissue has been characterized using various experimental techniques. Uniaxial tensile tests have been conducted at different strain rates on ex vivo human skin tissue samples excised from the back of cadavers to investigate the rate sensitivity of elastic modulus, ultimate tensile stress, and strain energy of human skin. It has been observed that these mechanical properties increase with higher strain rates and depend on the sample’s location and orientation with respect to the Langer lines within the skin^14–16^. The Poisson’s ratio of skin is found to depend on the orientation of collagen fibers in the skin region in stress relaxation experiments on in vivo human skin, and it increases during the relaxation phase^4^. The linear modulus of skin tissue correlates with the orientation intensity of the underlying collagen fibers in bulge tests, and an empirical relation has been developed to describe the variation of the skin modulus with orientation^17^. The viscoelastic properties of in vivo human forearm skin have proven useful in identifying the severity of various skin conditions, such as scleroderma, acromegaly, and Ehlers-Danlos syndrome^18^.

A Mooney-Rivlin-based five-term quasi-linear viscoelastic model is used to accurately describe the change in stress during stress relaxation tests of ex vivo skin samples at different strain rates. The effect of strain rate is found to be different for the five relaxation moduli and time constants of the five-term model^19^. The viscoelastic behavior of in vivo human skin has been described by the Kelvin-Voigt spring-damper model. Dynamic indentation tests on in vivo human skin using a novel device revealed that the damping coefficient of skin based on the Kelvin-Voigt model does not change with forcing frequency between 10 and 60 Hz^20^. The effect of hydration level and body mass index of the samples on the viscoelastic properties of skin are also studied using the novel device^20^. Several novel devices have been designed to assess the dynamic response of in vivo human skin for potential clinical applications in patient diagnosis and treatment related to skin conditions^20,21^. Pissarenko and Meyers^22^ offer an extensive review of experiments and constitutive material models examining the mechanical behavior of skin. However, these models are limited to the behavior of unburn skin tissue.

Thermal injury has a significant impact on the microstructure of skin tissue. When the skin tissue is heated beyond 65 °C, the collagen undergoes denaturation, transforming its protein from an organized crystalline structure to a random gel-like state^5^. Additionally, the rate of water loss from the skin surface increases significantly with temperature in live humans^23^. Biaxial testing of skin tissue under thermal treatment up to 95 °C for duration up to 5 min has resulted in shrinking of the sample anisotropically along the direction of principal orientation of collagen fiber. At high exposure times, skin samples couldn’t be tested due to severe softening of the sample from the denaturation of the collagen^8^. The elevation in temperature disrupts the lipid structure and keratin network along with the decomposition of keratin in the stratum corneum of the skin tissue. Consequently, the permeability of the skin increases by several folds at temperatures up to 150°C, by one to two orders of magnitude for heating up to 250°C, and by three orders of magnitude for heating above 300 °C^6^. These microstructural effects significantly alter the viscoelastic and rate dependent behavior of the burn skin tissue. Ex vivo porcine skin tissue exhibits a softening behavior as temperature increases^7^. Computational thermomechanical models have been developed to predict the effect of collagen denaturation on the anisotropic mechanical behavior of murine skin heated up to 95°C under biaxial tensile tests. The kinetics of collagen denaturation are described using a two-state model based on coiling of collagen fibers and transition to viscoelastic behavior, and a three-state model based on the sequence of fiber coil and fiber damage^24^. These studies are limited to the behavior of animal models for human skin. In stress relaxation tests, the elastic fraction, defined as the ratio of steady-state stress to peak stress, is found to be significantly higher in normal human skin tissue compared to hypertrophic scar tissue^25^. However, these studies involve skin tissues that are excised years after the burn injury. There is no research on the strain rate sensitivity of mechanical properties of full-thickness burn human skin tissue within hours of thermal injury.

The present study investigates the rate-dependent mechanical behavior of full-thickness burn ex vivo human skin tissue. Uniaxial tensile tests are carried out on the burn skin samples at a quasi-static loading rate of 0.3 mm/s and at rates of 2 mm/s and 8 mm/s, typical during surgery^26^. The stress–strain data collected from the uniaxial tests are utilized to estimate mechanical properties, ultimate tensile stress, ultimate tensile strain, and toughness, and two parameters of the Veronda–Westmann hyperelastic material model^27^. The similarity of the mechanical properties at the three rates is studied using univariate hypothesis tests and multivariate statistical analysis to evaluate the rate-dependent behavior of the full thickness burn human skin tissue.

The paper is organized as follows. Materials and methods section describes the experimental setup for the uniaxial tensile tests, along with the details of the univariate and multivariate statistical analyses. The statistical analysis results are presented in Results section﻿, followed by a discussion of the findings in Discussion section﻿. Conclusion section provides a summary of the study and outlines future directions. Appendix A includes the demographic information of the human skin samples and a comparison of various material models used to fit the experimental stress–strain data. The details of the univariate and multivariate statistical analyses are presented in Appendix B.

## Materials and methods

The mechanical behavior of full-thickness burn ex vivo skin samples is characterized under strain rates relevant to burn care surgery. We focus on mechanical properties, i.e., ultimate tensile stress, ultimate tensile strain, and toughness, and the parameters of Veronda–Westmann hyperelastic material model^27^. Univariate and multivariate statistical analyses are performed to study the rate-dependent behavior of the mechanical properties.

Sample collection section provides details of the burn human skin samples used in the study, and Uniaxial tensile test protocol section presents the experimental protocol for conducting uniaxial tensile tests on the burn skin samples. The procedure for calculating the material properties using data from the uniaxial tensile tests is outlined in Characterization of material properties section. Additionally, in Statistical analysis section, we provide details about the statistical analysis methods employed in this study.

### Sample collection

The debrided skin samples were collected from patients with full or deep-partial thickness burn injuries at the Harborview Medical Center Burn Unit in Seattle, WA. All methods and procedures were carried out in accordance with the protocol, and the relevant guidelines and regulations approved by the University of Washington (UW) Institutional Review Board (IRB) (IRB ID: STUDY00008694). The UW IRB committee waived the informed consent requirement for the access and use of medical records and debrided skin tissue specimens.

A total of 320 burn skin samples were obtained from 15 patients (13 males and 2 females) within an age range of 38 to 78 years. The time between the burn injury and the debridement of the skin ranged from 23 to 68 h, except for four extreme cases when burn skin was debrided after 11, 120, 139, and 360 h. The anatomical location of the burn injury for all the subjects is provided in Appendix A. The debrided skin samples were submerged in 1X phosphate-buffered saline (PBS) (pH 7.4) to prevent loss of hydration and stored in a refrigerator. The samples were then transferred to the University of Washington for testing.

### Uniaxial tensile test protocol

Uniaxial tensile tests were conducted on debrided and discarded human skin tissues until specimen rupture. The tests were carried out at the University of Washington in Seattle, WA within 72 h of excision, following the experimental protocol described elsewhere^28^. A summary of the protocol is presented below.

**Figure 1:**
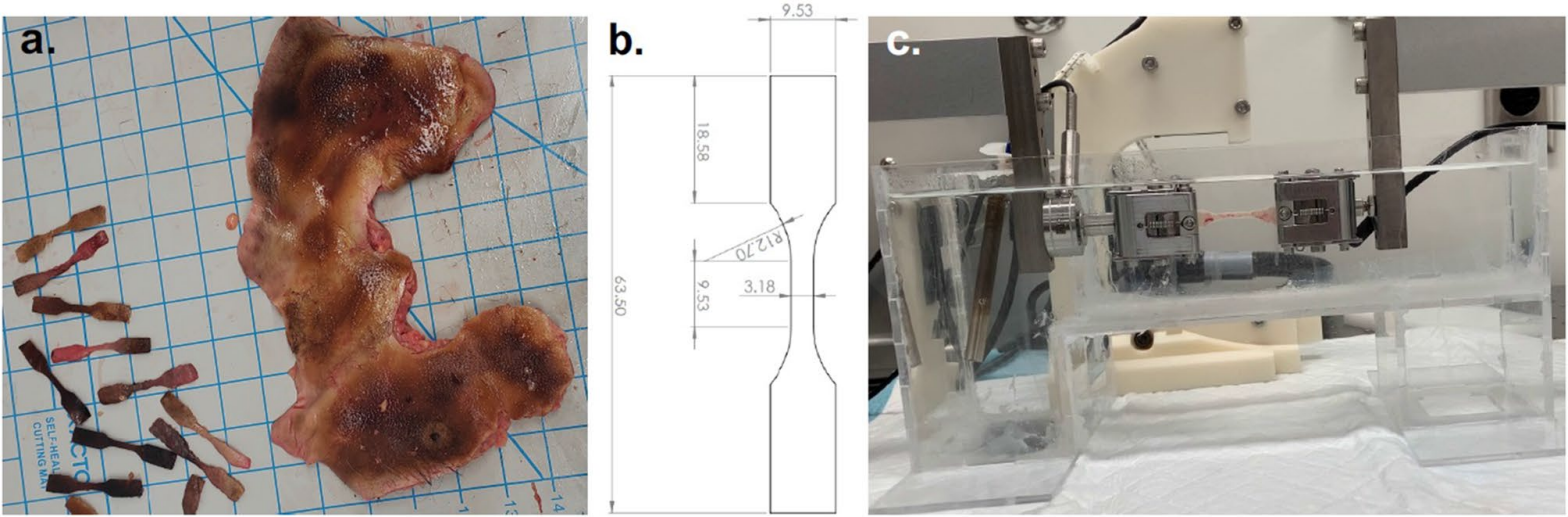
(**a**) The dog-bone shape skin samples are shown on the left and debrided or discarded full-thickness burn human skin is shown on the right. (**b**) The dimensions of the dog-bone specimen created using the ASTM D638 Type V die. (**c**) The experimental set up for the uniaxial tensile test of dog-bone shape full-thickness burn human skin sample held between the grips and fixtures in a 37 °C PBS bath in a transparent container using TA Instruments ElectroForce TestBench.

After removing the subcutaneous tissue of the skin samples, an ASTM D638 Type V die was used to create standard dog-bone shape specimens (Fig. 1a) for the testing. The subcutaneous fat or hypodermis was removed since it does not contribute towards the mechanical behavior of skin tissue^7,11,29,30^. The dimensions of the dog-bone specimen are shown in Fig. 1b. The uniaxial tensile tests were performed at loading rates of 0.3 mm/s, 2 mm/s, and 8 mm/s until specimen failure. The tests were conducted using a TA Instruments ElectroForce TestBench (TA Instruments, MN; max. velocity of 3.2 m/s) instrument in a 37°C PBS bath, as shown in Fig. 1c. The TestBench holds the sample in place without slipping using self-tightening spring grips. The motors on the TestBench are on a track that eliminates bending of the sample. Force and displacement measurements were recorded to evaluate the mechanical properties of the samples. The uniaxial tensile testers have been extensively used to measure force and displacement of skin tissue samples in previous studies^29–32^. Errors in displacement measured by the software of the TestBench based on the displacement between the grips, may be neglected since the strain rates of the current study are small (< 1/s). A total of 107, 105, and 108 burn skin samples were tested at the loading rates of 0.3 mm/s, 2 mm/s, and 8 mm/s, respectively. The mean thickness of the skin samples was 2.1 ± 1.0 mm.

### Characterization of material properties

**Figure 2:**
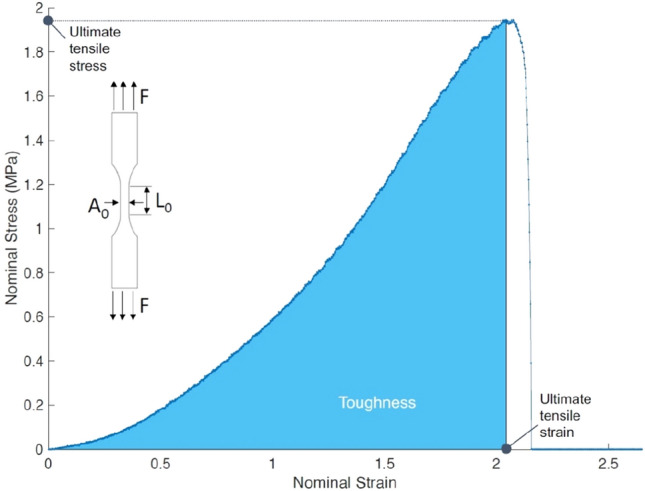
A typical nominal stress–strain curve of the full-thickness burn ex vivo human skin tissue sample undergoing the uniaxial tensile test. The applied force (*F*), initial length (*L*_0_), and initial cross-section area (*A*_0_) of the *dog bone* sample are shown in the inset.

The force ($F$) and displacement ($\Delta L$) measurements recorded during the uniaxial tensile tests of the dog bone samples are used to compute the nominal stress ${\sigma }_{N}=F/{A}_{0}$ and nominal strain ${\varepsilon }_{N}=\Delta L/{L}_{0}$, where ${A}_{0}$ is the initial cross-sectional area of the gauge section and ${L}_{0}$ is the initial length of the sample (Fig. 2). The ultimate tensile stress, ultimate tensile strain, and toughness are estimated from the nominal stress–strain curve, as indicated in Fig. 2. The ultimate tensile stress and strain describe the maximum nominal stress and strain at the onset of failure. The area under the nominal stress–strain curve is computed to estimate the toughness of the sample.

The Veronda–Westmann hyperelastic material law is used to describe the stress–strain response of the full-thickness burn human skin tissues. The strain energy density function is given as

1 $$\Psi \left({\overline{I} }_{1},{\overline{I} }_{2}\right)=\frac{\mu }{\gamma }\left({e}^{\gamma \left({\overline{I} }_{1}-3\right)}-1\right)-\mu \left({\overline{I} }_{2}-3\right),$$

where $\mu$ and $\gamma$ are two material coefficients, ${\overline{I} }_{1}={\lambda }_{U}^{2}+\frac{2}{{\lambda }_{U}}$ and ${\overline{I} }_{2}=2{\lambda }_{U}+\frac{1}{{\lambda }_{U}^{2}}$ are the first and second deviatoric strain invariants, respectively. The principle stretch along the uniaxial loading direction is given as ${\lambda }_{U}=1+{\varepsilon }_{N}$, where ${\varepsilon }_{N}$ is the nominal strain under uniaxial loading conditions. For the uniaxial tensile tests, the nominal stress for the Veronda–Westmann material is

2 $${\sigma }_{N}=2\mu \left(1-{\lambda }_{U}^{-3}\right)\left({\lambda }_{U}{e}^{\gamma \left({\overline{I} }_{1}-3\right)}-1\right).$$

Nominal or engineering stress and strain have been used in the literature for modeling nonlinear mechanical behavior of soft tissues^7,27,29–32^. The Veronda–Westmann model was developed to model the mechanical response of ex vivo cat skin under uniaxial tensile loading^27^. For the full-thickness burn human skin tissues, the model fits the experimental stress–strain data with goodness-of-fit measure, *R*^2^ = 0.99 for each of the three loading rates. Hence, the Veronda–Westmann model is deemed appropriate to describe the stress–strain constitutive response of full-thickness burn human skin tissues in this study. A comparison of Veronda–Westmann model with other hyperelastic material laws typically used for soft tissue modeling is presented in Appendix A. The least-squares method is used to estimate the parameters of the material laws.

### Statistical analysis

The methodology for the univariate and multivariate statistical analyses is presented in Univariate statistical analysis and Multivariate statistical analysis sections, respectively.

#### Univariate statistical analysis

The distributions of the five material parameters of the full-thickness burn human skin tissue samples were compared among the three loading rates. The details of the univariate hypothesis tests performed in the study can be found elsewhere^28^. A brief summary is provided here. The Shapiro–Wilk normality test^33^ was applied initially on sample data of each material parameter at each loading rate. If a pair of sample data for a material parameter at two rates were drawn from the normal distribution, the two-sample *F*-test for equal variances^34^ and the two-sample *t*-test for equal or unequal variances^34^ were applied. The result of the two-sample *t-*test for equal or unequal variances would indicate the similarity between the material parameter at the two rates. Otherwise, the Kolmogorov–Smirnov test^35^ was applied on the pair of sample data. The Wilcoxon rank-sum test^34^ or the two-sample *t*-test for unequal variances^36^ was applied, if the two samples were drawn from continuous distributions with same or different shapes, respectively. The result of the Wilcoxon rank-sum test or the two-sample *t-*test for unequal variances would indicate the similarity between the material parameter at the two rates. For all hypothesis tests, the significance was taken as 0.01.

In order to verify the minimum sample size required for the study, the Cohen’s d statistic is calculated in a post-hoc analysis to estimate the effect size for a sample pair. This statistic is a numerical representation of the strength of the relationship between two variables in a population. An effect size (d) of 0.2, 0.5, and 0.8 can be regarded as small, medium, and large, respectively^37^. The sample size estimations were calculated using G*Power 3.1.9.7^38^, with the significance level and power taken as 0.05 and 0.85, respectively.

#### Multivariate statistical analysis

A rejection of null hypothesis using univariate hypothesis tests underscores the difference between two classes based on their mean or median. However, it does not provide the essential qualitative insight into the significance or the degree of such differences between the classes. Hence, a multivariate analysis^39^ is performed to classify the samples loaded at various rates using a feature set of three mechanical properties, i.e., ultimate tensile stress and strain, and toughness, and the two parameters, μ and γ, of the Veronda–Westmann hyperelastic material model.

The multiclass classification was conducted for full-thickness burn human skin specimens, utilizing samples obtained from the three loading rates. Logistic regression^40^ was employed for the classification task. To ensure an independent assessment of the classifier, a leave-one-out cross-validation^41^ was implemented. The degree of distinction among the three loading rates is inferred from the misclassification error, indicated by the percentage of incorrectly classified data points or samples.

## Results

To examine the rate-dependent mechanical behavior of full-thickness burn human skin tissues, univariate and multivariate statistical analyses were conducted using five material parameters: ultimate tensile stress, ultimate tensile strain, toughness, and the parameters of the Veronda–Westmann material model, which were estimated based on experimental data.

The results of the univariate and multivariate analysis are presented in Univariate hypothesis testing and Multivariate classification sections, respectively.

### Univariate hypothesis testing

**Figure 3:**
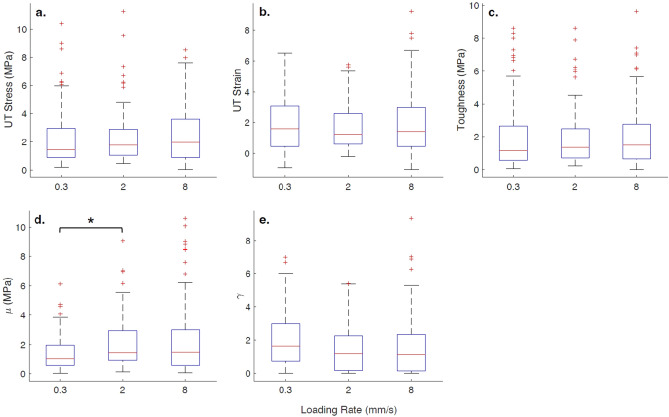
The box plots of (**a**) ultimate tensile stress (UT stress), (**b**) ultimate tensile strain (UT strain), (**c**) toughness, and parameters (**d**) μ and (**e**) γ of the Veronda–Westmann model for the full-thickness burn human skin tissue at loading rates of 0.3 mm/s, 2.0 mm/s, and 8.0 mm/s. The ‘*’ indicates a significant difference in the Wilcoxon rank-sum test.

Samples of the full-thickness burn human skin tissue with at least one of the five parameters exceeding three times the interquartile range of the distribution of the parameter were removed from the analysis. This is acceptable in statistical analysis, and it doesn’t introduce any bias in the results^42^. The number of samples used in the statistical analysis after removing the outliers for the 0.3 mm/s, 2.0 mm/s, and 8.0 mm/s loading rates is 95, 92, and 102, respectively. The Cohen’s d effect size for the human skin tissue samples based on the five material parameters is 0.4, and the allocation ratio is chosen to be 1, which is the ratio found in the study. The minimum sample size of human tissues for each loading rate, based on the Cohen’s d value and the allocation ratio, is 91, confirming that the sample sizes used in this study are appropriate for the univariate hypothesis tests. The box plots of the material properties of the human skin tissue at the three loading rates are shown in Fig. 3. Outliers are typically observed only towards the 75th percentile in the distribution for the mechanical properties in Fig. 3 due to the lower limit of 0.0 on the properties that would not allow for negative outliers. The large range in mechanical properties is typical for skin tissues^22^. The median, 25th, and 75th percentile values for the five material parameters based on samples loaded at all three rates are provided in Table 1.

**Table 1:** Median, 25th, and 75th percentile values for the five material parameters based on samples loaded at all three rates.

Material parameter	Median	25th Percentile	75th Percentile
UT stress (MPa)	1.73	0.97	3.20
UT strain	1.69	1.27	2.47
Toughness (MPa)	1.38	0.64	2.65
μ (MPa)	0.32	0.17	0.66
γ	0.07	0.01	0.12

The results of the univariate hypothesis tests are summarized in Appendix B. The Shapiro–Wilk test showed that the full-thickness burn human skin samples have not been sampled from a normal distribution for each loading rate of 0.3 mm/s, 2 mm/s, and 8 mm/s, based on any of the five mechanical parameters. The Kolmogorov–Smirnov test indicated that the skin samples loaded at each of the three rates belong to the same continuous distribution (not a normal distribution), based on the five parameters. Hence, the Wilcoxon rank-sum test is conducted to evaluate the similarity between each of the five mechanical parameters at any two rates, based on the median of the parameter distribution. It is found that we cannot reject the hypotheses stating that the medians of ultimate tensile stress, ultimate tensile strain, toughness, and the parameter γ of the Veronda–Westmann material model are equal for each loading rate. Hence, it can be concluded that these properties are equal for all three loading rates. This indicates that these mechanical properties of the full-thickness burn human skin are independent of the loading rate. Additionally, Fig. 3 provides supporting evidence that the median and ranges of these properties of the burn skin are not distinctively different.

However, there is one notable exception. The hypothesis stating that the median of the parameter μ of the material model is equal at 0.3 and 2 mm/s, is rejected when the Wilcoxon rank-sum test is applied. However, when applying the same test, we cannot reject the null hypotheses that the medians of the parameter μ are equal between the loading rates of 0.3 and 8 mm/s as well as between the rates of 2 and 8 mm/s. Therefore, the difference in the medians of the parameter μ between 0.3 and 2 mm/s might be negligibly small, although it is statistically significant. In other words, the parameter μ shows dependance on the loading rate for samples loaded at 0.3 and 2 mm/s, although it is found to be independent of the loading rate for samples loaded at 0.3 and 8 mm/s or for samples loaded at 2 and 8 mm/s. Hence, the rate dependent behavior of the parameter μ of the material model might be negligibly small.

### Multivariate classification

The multivariate analysis using logistic regression statistical model was carried out to differentiate the five material parameters for full-thickness burn human skin tissues. Multiclass classification was performed to distinguish the tissue specimens loaded at the three rates, i.e., 0.3 mm/s, 2 mm/s, and 8 mm/s. The confusion matrix obtained from the leave-one-out cross-validation is provided in Appendix B. The multiclass performance metrics computed from the confusion matrix are as follows. The accuracy is 0.3910, Matthew's correlation coefficient (MCC)^43^ is 0.0851, Fowlkes-Mallow’s index (FMI)^44^ is 0.3429, and the Adjusted Rand index (ARI)^45^ is 0.0032.

**Figure 4:**
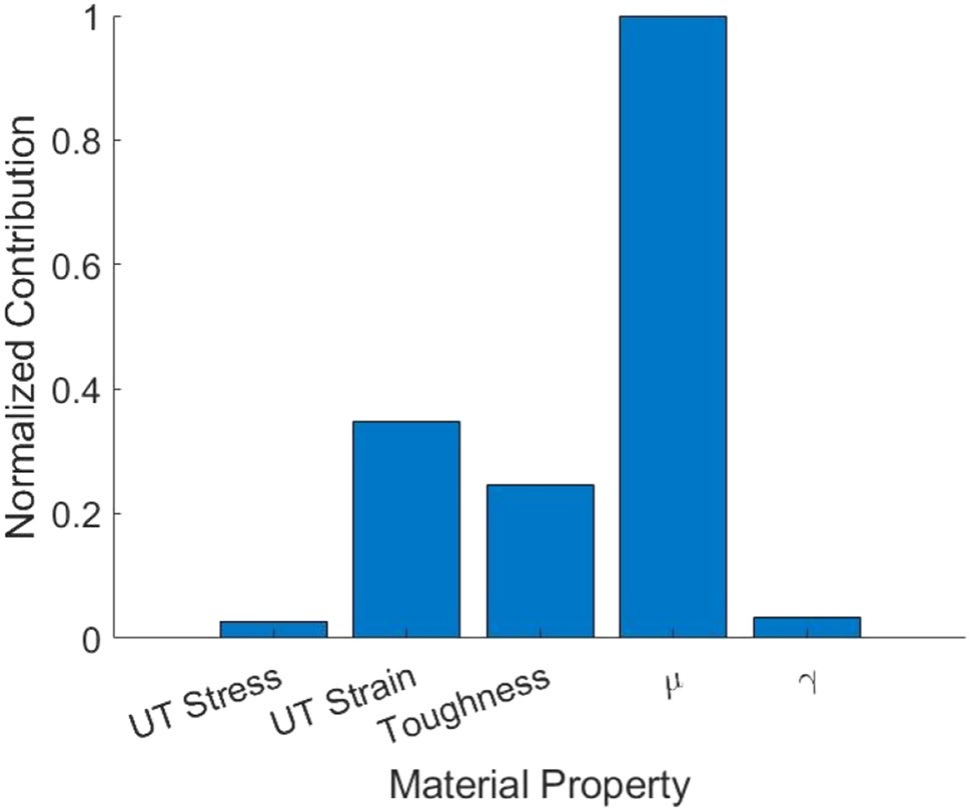
Contribution of each material property in the multiclass classification of full-thickness burn human skin tissue samples into the three loading rate classes of 0.3 mm/s, 2 mm/s, and 8 mm/s.

The results indicate that the full-thickness burn human skin tissue samples at the three loading rates are not separable with a satisfactory degree of accuracy (< 0.4) based on the five mechanical parameters. This finding is further supported by the low value of the FMI and the negligible values of MCC and ARI metrics. A factor analysis of the classifier is performed to quantify the contribution of each mechanical property in differentiating the samples among the three loading rates. This analysis aims to assess the influence of each of the five material parameters in distinguishing the samples loaded at the three rates. Figure 4 shows that the parameter μ of the material model is the most discriminative factor among the five parameters, while the ultimate tensile stress and material model parameter γ are the least discriminative factors. This observation is consistent with the results obtained from the univariate hypothesis tests, where the hypothesis that the median of the parameter μ is equal for the loading rates of 0.3 and 2 mm/s was rejected.

## Discussion

The univariate and multivariate statistical analyses using mechanical properties, i.e., ultimate tensile stress, ultimate tensile strain, and toughness, along with the parameters of the Veronda–Westmann hyperelastic material law reveal that the full thickness burn human skin tissue is rate independent. This finding contrasts with the known behavior of skin tissue, even at elevated temperatures^7^.

The rate-dependent or viscoelastic behavior of human skin tissue is mainly attributed to the shear interaction between collagen and the hydrated matrix of proteoglycans in the skin tissue^13^. The proteoglycans possess a central protein core covalently bonded to the glycosaminoglycan (GAG) chains. The highly hydrophilic GAGs combine with water molecules in the matrix to form gelatin-like structures, contributing to the viscous properties of the skin tissue^46^. In a previous study, Raman spectrum analysis of porcine skin tissue indicated that the water band in protein secondary structures, dominated by the bending of the –OH bond^47–49^, is entirely absent in the full-thickness burn skin samples^50^. This indicates a weakening of the hydrogen bonds between the amino acid groups in tropocollagen molecule in the burn skin^50^. The viscosity of collagen fibrils in skin tissue is proportional to the rate of breaking and formation of hydrogen bonds during molecular interactions within the fibrils^51,52^. Hence, the weakening of hydrogen bonds and its impact on viscosity and rate-dependent behavior of the burn human skin needs to be further investigated.

Normal skin is anisotropic and its mechanical behavior depends on the anatomical location due to variation in collagen fiber orientation at various anatomical locations of the skin^22^. In this work, the effect of anatomical location has not been studied due to limited availability of the debrided human skin samples obtained from various anatomical locations. The anisotropic mechanical behavior has also been observed in murine skin tissue samples heated at 95°C^8^. However, the full thickness burns are induced when skin is exposed to high temperatures (> 232°C) for longer duration (> 10s)^28^. At such high temperatures, the collagen fibers transform from an organized crystalline structure to a random gel-like state due to denaturation^53^. Hence, the mechanical behavior of the full thickness burn human skin tissue may not exhibit significant dependance on the collagen fiber orientation. However, the effects of anisotropy and anatomical location on the rate dependent mechanical behavior of the full thickness burn human skin can be further investigated.

This study is limited by the ex vivo uniaxial testing of the burn skin tissues. Novel in vivo testing protocols need to be developed to include the effect of physiological phenomena, such as blood perfusion, pre-stress, muscle activation, etc., on the mechanical behavior of burn skin. Ultrasound based devices may be explored for in vivo mechanical characterization. It should be noted that the rate independent mechanical behavior of full thickness burn human skin is observed for loading rates between 0.3 to 8 mm/s. Further study will be required to investigate the mechanical behavior of burn human skin beyond these loading rates. No-contact displacement measurement techniques can be used for higher accuracy^4^. The present study can be extended to investigate changes in the mechanical behavior of human skin during wound healing following thermal injury.

The findings of this work hold significant importance in the study of burn skin under dynamic loads and in various medical applications involving thermal treatment of the skin.

## Conclusion

The rate-dependent mechanical behavior of debrided full-thickness burn human skin was investigated through the evaluation of mechanical properties, i.e., ultimate tensile stress, ultimate tensile strain, and toughness, and the parameters of Veronda–Westmann hyperelastic material law. The mechanical properties were estimated from the force–displacement data obtained during uniaxial tensile testing of skin samples at strain rates relevant to burn care surgery, specifically, 0.3 mm/s, 2.0 mm/s, and 8.0 mm/s. Univariate and multivariate statistical analyses were conducted on the mechanical properties at three loading rates to examine their rate-dependent behavior. The results of the univariate hypothesis tests demonstrated that the distributions of all properties were equal. Further, when employing a logistic regression-based multivariate classifier, the samples loaded at the three different rates could not be distinguished. Consequently, full-thickness burn human skin exhibited rate-independent mechanical behavior, which contradicts the expected behavior of biological soft tissues. This finding is of considerable significance, shedding new light on the unique, rate-independent behavior of full thickness burn skin.

### Supplementary Information

Supplementary Information.

## Data Availability

The datasets generated during and/or analyzed during the current study are available from the corresponding author on reasonable request.
