# Supplementary material for: Mechanical behavior of full-thickness burn human skin is rate-independent
Source: Sci Rep. 2024 May 15;14:11096. doi: 10.1038/s41598-024-61556-8 (PMC11096406; doi:10.1038/s41598-024-61556-8)
Supplement: Supplementary file 1 — Supplementary Information. [file 41598_2024_61556_MOESM1_ESM.docx]

**Supplementary Information**

**Mechanical behavior of full-thickness burn human skin is rate-independent**

Samara Gallagher,^1,2^ Kartik Josyula,^2^ Rahul*,^2^ Uwe Kruger,^2,3^ Alex Gong,^4^ Agnes Song,^4^ Emily Eschelbach,^5^ David Crawford,^5^ Tam Pham,^5^ Robert Sweet,^4^ Conner Parsey,^6^ Jack Norfleet,^6^ Suvranu De,^1,2,3^

^1^ Department of Mechanical, Aerospace, and Nuclear Engineering

^2^ Center for Modeling, Simulation and Imaging in Medicine

^3^ Department of Biomedical Engineering

Rensselaer Polytechnic Institute, Troy NY

^4^ Center for Research in Education and Simulation Technologies

^5^ UW Medicine Regional Burn Center at Harborview Medical Center

University of Washington, Seattle WA

^6^ U.S. Army Combat Capabilities Development Command - Soldier Center, Simulation and Training Technology Center, Orlando FL

**Appendix A**

**A.1. Anatomical location of burns in human skin samples**

The anatomical location of the burn injury in the debrided/discarded full-thickness (or deep partial-thickness) burn human skin tissues for the 15 subjects and the number of dog bone samples obtained from the subject are given in Table A.1.

**Table A.1**. Anatomical location of the burn injury of the human subjects.

| **Subject No.** | **Anatomical location of burn injury** | **Number of samples** | | |
| --- | --- | --- | --- | --- |
|  |  | **0.3 mm/s** | **2 mm/s** | **8 mm/s** |
| 1 | Right upper extremity | 1 | 1 | 1 |
| 2 | Right arm | 0 | 0 | 2 |
| 3 | Left flank and Left arm | 9 | 9 | 11 |
| 4 | Left and right thighs | 8 | 9 | 9 |
| 5 | Left and right arms and Chest | 10 | 9 | 9 |
| 6 | Flank | 2 | 2 | 2 |
| 7 | Right forearm | 2 | 2 | 1 |
| 8 | Abdomen | 3 | 3 | 3 |
| 9 | Chest | 4 | 3 | 4 |
| 10 | Left thigh (posterior) | 7 | 7 | 7 |
| 11 | Bilateral calf, thigh, arm, and flank | 34 | 33 | 32 |
| 12 | Right arm, flank, and thigh | 10 | 10 | 10 |
| 13 | Right thigh to toes, Left shin to toes | 8 | 8 | 8 |
| 14 | Right flank and shoulder (thorax) | 7 | 7 | 7 |
| 15 | Right buttock | 2 | 2 | 2 |

**A.2. Comparison of hyperelastic material laws**

The Veronda-Westmann hyperelastic material model [1] is compared with the Arruda-Boyce material model [2] and the reduced second order polynomial model [3]. These material models are fit to the experimental nominal stress-strain data using the least squares method. A typical curve fit for the three models is shown in Figure A.1 for the burn human skin tissue samples for each loading rate, i.e., 0.3 mm/s, 2 mm/s, and 8 mm/s. The Veronda-Westmann material model has the best fit to the data with the R^2^ goodness-of-fit measure of 0.99 for all three loading rates. Hence, the Veronda-Westmann model is used in the present study to describe the stress-strain response of the full thickness burn human skin tissues at various loading rates.

**Figure A.1**. Curve-fitting of the Arruda-Boyce model, reduced second order polynomial model, and the Veronda-Westmann model to the experimental nominal stress-strain data from the uniaxial tensile tests on full thickness burn human skin tissue loaded at (a) 0.3 mm/s, (b) 2 mm/s, (c) 8 mm/s.

**Appendix B**

**B.1. Univariate hypothesis tests**

The results from the Shapiro-Wilk normality test of the five material parameters at the three loading rates for human skin tissues are given in Table B.1. The univariate statistical analysis of the comparison of the material parameters of the full-thickness burn human skin tissue between any two loading rates is given Table B.2.

**Table B.1**. The *p*-value of the Shapiro-Wilk normality test of the five material parameters of human skin tissues at three loading rates.

| **Material Parameter** | **Loading Rate** | ***p*-Value** | **Normal Distribution?** |
| --- | --- | --- | --- |
| UT Stress | 0.3 mm/s | 0.0 | No |
|  | 2 mm/s | 0.0 | No |
|  | 8 mm/s | 0.0 | No |
| UT Strain | 0.3 mm/s | 0.0001 | No |
|  | 2 mm/s | 0.0 | No |
|  | 8 mm/s | 0.0 | No |
| Toughness | 0.3 mm/s | 0.0 | No |
|  | 2 mm/s | 0.0 | No |
|  | 8 mm/s | 0.0 | No |
| μ | 0.3 mm/s | 0.0 | No |
|  | 2 mm/s | 0.0 | No |
|  | 8 mm/s | 0.0 | No |
| γ | 0.3 mm/s | 0.0 | No |
|  | 2 mm/s | 0.0 | No |
|  | 8 mm/s | 0.0 | No |

**Table B.2**. The *p*-value of univariate statistical tests to compare the five material parameters of human skin tissues at any two loading rates.

| **Material Parameter** | **Loading Rate** | **Statistical Test** | ***p* Value** | **Conclusion** |
| --- | --- | --- | --- | --- |
| UT Stress | 0.3 & 2 mm/s | Kolmogorov-Smirnov |  | Same distribution |
|  |  | Wilcoxon rank-sum | 0.3632 | Equal medians |
|  | 2 & 8 mm/s | Kolmogorov-Smirnov |  | Same distribution |
|  |  | Wilcoxon rank-sum | 0.5863 | Equal medians |
|  | 0.3 & 8 mm/s | Kolmogorov-Smirnov |  | Same distribution |
|  |  | Wilcoxon rank-sum | 0.2276 | Equal medians |
| UT Strain | 0.3 & 2 mm/s | Kolmogorov-Smirnov |  | Same distribution |
|  |  | Wilcoxon rank-sum | 0.5731 | Equal medians |
|  | 2 & 8 mm/s | Kolmogorov-Smirnov |  | Same distribution |
|  |  | Wilcoxon rank-sum | 0.8009 | Equal medians |
|  | 0.3 & 8 mm/s | Kolmogorov-Smirnov |  | Same distribution |
|  |  | Wilcoxon rank-sum | 0.8307 | Equal medians |
| Toughness | 0.3 & 2 mm/s | Kolmogorov-Smirnov |  | Same distribution |
|  |  | Wilcoxon rank-sum | 0.2852 | Equal medians |
|  | 2 & 8 mm/s | Kolmogorov-Smirnov |  | Same distribution |
|  |  | Wilcoxon rank-sum | 0.7890 | Equal medians |
|  | 0.3 & 8 mm/s | Kolmogorov-Smirnov |  | Same distribution |
|  |  | Wilcoxon rank-sum | 0.2999 | Equal medians |
| μ | 0.3 & 2 mm/s | Kolmogorov-Smirnov |  | Same distribution |
|  |  | Wilcoxon rank-sum | 0.0013 | Unequal medians |
|  | 2 & 8 mm/s | Kolmogorov-Smirnov |  | Same distribution |
|  |  | Wilcoxon rank-sum | 0.2862 | Equal medians |
|  | 0.3 & 8 mm/s | Kolmogorov-Smirnov |  | Same distribution |
|  |  | Wilcoxon rank-sum | 0.1172 | Equal medians |
| γ | 0.3 & 2 mm/s | Kolmogorov-Smirnov |  | Same distribution |
|  |  | Wilcoxon rank-sum | 0.0341 | Equal medians |
|  | 2 & 8 mm/s | Kolmogorov-Smirnov |  | Same distribution |
|  |  | Wilcoxon rank-sum | 0.7614 | Equal medians |
|  | 0.3 & 8 mm/s | Kolmogorov-Smirnov |  | Same distribution |
|  |  | Wilcoxon rank-sum | 0.0755 | Equal medians |

**B.2. Multivariate classification**

The multivariate analysis using logistic regression statistical model was carried out to differentiate the five material parameters for full-thickness burn human skin tissues between the three rates, i.e., 0.3 mm/s, 2 mm/s, and 8 mm/s. The confusion matrix obtained from the leave-one-out cross-validation is provided in Table B.3.

**Table B.3**. Confusion matrix for multiclass classification of burn human skin tissues at three loading rates, obtained from leave-one-out cross-validation.

|  | | Predicted Loading Rate | | |
| --- | --- | --- | --- | --- |
|  |  | 0.3 mm/s | 2 mm/s | 8 mm/s |
| Actual Loading Rate | 0.3 mm/s | 45 | 22 | 28 |
|  | 2 mm/s | 34 | 25 | 33 |
|  | 8 mm/s | 41 | 18 | 43 |

**References**

[1] Veronda, D. R., and Westmann, R. A., 1970, “Mechanical Characterization of Skin—Finite Deformations,” J. Biomech., **3**(1), pp. 111–122.

[2] Arruda, E. M., and Boyce, M. C., 1993, “A Three-Dimensional Constitutive Model for the Large Stretch Behavior of Rubber Elastic Materials,” J. Mech. Phys. Solids, **41**(2), pp. 389–412.

[3] Rivlin, R. S., and Saunders, D. W., 1951, “Large Elastic Deformations of Isotropic Materials VII. Experiments on the Deformation of Rubber,” Philos. Trans. R. Soc. London. Ser. A, Math. Phys. Sci., **243**(865), pp. 251–288.
